# State of the Art of Stimuli-Responsive Liposomes for Cancer Therapy

**Published:** 2017

**Authors:** Elmira Heidarli, Simin Dadashzadeh, Azadeh Haeri

**Affiliations:** a *Department of Pharmaceutics, School of Pharmacy, Shahid Beheshti University of Medical Sciences, Tehran, Iran. *; b *Protein Technology Research Center, Shahid Beheshti University of Medical Sciences, Tehran, Iran.*

**Keywords:** Liposomes, Cancer, Stimuli-responsive, Thermosensitive, Magnetoliposomes, Light, Ultrasound, pH, Redox, Enzyme

## Abstract

Specific delivery of therapeutic agents to solid tumors and their bioavailability at the target site are the most clinically important and challenging goals in cancer therapy. Liposomes are promising nanocarriers and have been well investigated for cancer therapy. In spite of preferred accumulation in tumors via the enhanced permeability and retention (EPR) effect, inefficient drug release at the target site and endosomal entrapment of long circulating liposomes are very important obstacles for achieving maximum anticancer efficacy. Thus, additional strategies such as stimulus-sensitive drug release are necessary to improve efficacy. Stimuli-sensitive liposomes are stable in blood circulation, however, activated by responding to external or internal stimuli and control the cargo release at the target site. This review focuses on state of the art of stimuli-responsive liposomes. Both external stimuli-responsive liposomes, including hyperthermia (HT), magnetic, light, and ultrasound-sensitive liposomes and internal stimuli (pH, reduction, and enzyme) responsive liposomes are covered.

## Introduction

Cancer is one of the most common causes of death in the world that was responsible for millions of deaths in the 20^th^ century, and still remains one of most challenging diseases to treat. Cancer as a significant cause of morbidity and mortality with more than ten million new cases every year is the biggest public health concern (1, 2). Although chemotherapeutics are widely used for cancer treatment, and effective to some extent, their nonspecific biodistribution to normal tissues and affecting healthy rapidly dividing cells (enterocytes, white blood cells, *etc.*) cause drug-induced toxicity and numerous serious side effects. In addition, free cancer therapeutics suffer from poor solubility, low stability, rapid *in-vivo* degradation, and short plasma residence time. To overcome the aforementioned limitations of chemotherapeutics, various nanoparticulate systems including liposomes, polymeric nanoparticles, polymeric micelles, dendrimers, and inorganic nanoparticles have been increasingly investigated for cancer therapy due to several advantages (3-7).

**Figure 1:**
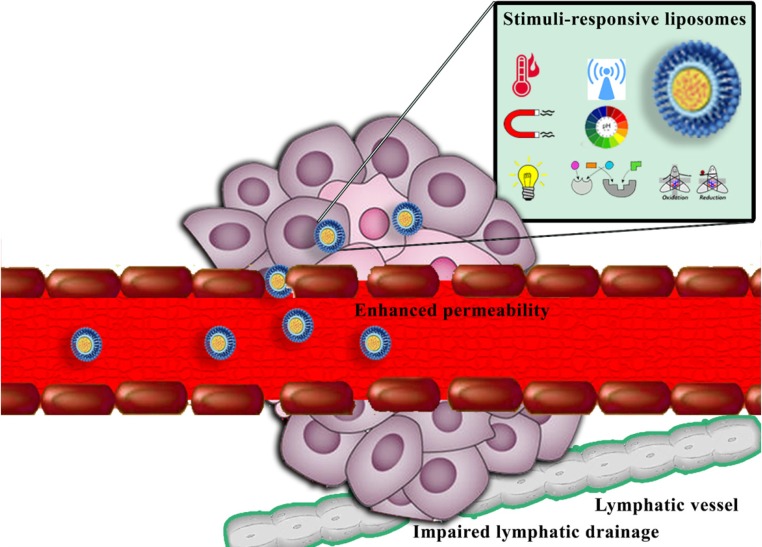
Schematic illustration of enhanced permeability and retention (EPR) effect and passive targeting of nanocarriers to solid tumors

The enhanced permeability and retention (EPR) effect, which is due to unique structural features of many solid tumors, including hypervasculature, poorly aligned defective endothelial cells lacking smooth muscle layer, and impaired lymphatic drainage (Figure 1), has the critical role in nanoparticles accumulation at the tumor site (8, 9). However, the effective nanocarriers of chemotherapeutics cannot only rely on the EPR effect. In order to use beneficial features of the EPR effect for anticancer delivery, nanomedicines should exhibit prolonged circulation time. Size, surface charge, hydrophobicity, composition, PEGylation, and shape are critical parameters in pharmacokinetic properties of nanostructures (10, 11).

Although nanoparticle accumulation in tumor site is very critical for antitumor efficacy enhancement and reduced adverse effects, inefficient drug release at the target site and endosomal entrapment of nanoparticles are very important obstacles for achieving maximum efficacy. To overcome these problems, stimuli-responsive nanocarriers are designed to trigger drug release by either externally applied stimuli (such as hyperthermia (HT), magnetic, light, and ultrasound) or pathophysiological characteristic of tumors (internal stimuli). Usually in tumors, the pH value is lower and reduction potential and enzyme activity are different. These features provide the opportunity to use these endogenous factors as triggers to control cargo release at the target sites (12-16).

**Figure 2:**
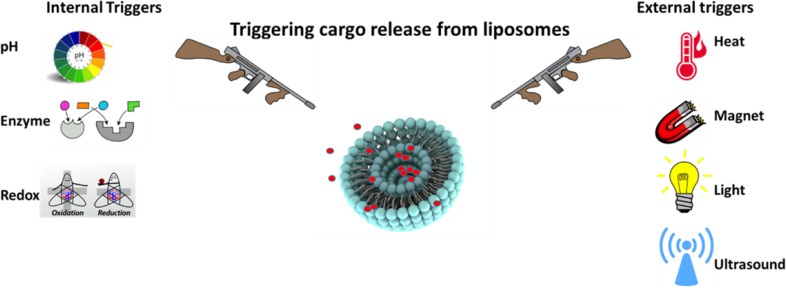
Schematic illustration of stimuli-responsive liposomes triggered upon external as well as internal stimulation

Among various nanoparticles, liposomes are widely-studied colloidal particles for cancer therapeutics delivery. Liposomes are bilayer vesicles composed of phospholipids and cholesterol and formed spontaneously when lipids are dispersed into an aqueous phase. Liposomes have numerous advantages such as biodegradability, excellent biocompatibility, non-immunogenicity, lack of toxicity, ability to incorporate hydrophilic and hydrophobic cargoes, enhanced bioavailability, and high stability that make them a unique carrier for drug and gene delivery (17-20). Beyond cancer, liposomes are used to enhance drug efficacy in various diseases including cardiovascular diseases (21-23), infections (24), autoimmune disorders (25), and skin diseases (26). In this article, we focus on recent studies of stimuli-sensitive liposomes for cancer therapy. Both external stimuli-responsive liposomes, including HT, magnetic, light, and ultrasound-sensitive liposomes and internal stimuli (pH, reduction, and enzyme) responsive liposomes will be covered (Figure 2).

*External stimuli-responsive liposomes*

*Thermosensitive liposomes*

Hyperthermia (HT) was mentioned as a cancer treatment throughout the Middle ages (27). However, unsatisfactory heating techniques and equipment, the lack of precise non-invasive thermometry, and ineffective targeting of deeply-seated tumors hampered clinical applications of HT (28). During the past two decades, testing HT as a component of cancer treatment strategies in a total of 109 trials has improved confidence in its clinical potential (29). The combination of mild HT with chemotherapy and radiation has been shown to improve cancer outcomes (30).

When combined with thermosensitive liposomes (TSL), HT can improve treatment efficacy by various mechanisms: (i) increasing liposomes accumulation in the tumor site by increasing tumor vascular permeability and local blood flow, (ii) triggering cargo release from TSL within tumor vasculature and interstitium, (iii) increasing cancer cell membrane permeability to the released drugs, and (iv) being directly cytotoxic to tumor cells (31, 32). Therefore, combination of HT and TSL administration holds great potential in cancer therapy (Table 1).

The design of TSL is based on pioneering work of Yatvin *et al.* (33) in 1978 on neomycin liposomal formulation which was the first formulation of traditional TSL. Over the next few decades, traditional TSL were further developed from lipid membranes that undergo phase transition from a gel to a liquid phase upon heating and the encapsulated cargoes leak out of the liposome during the phase transition. Early formulations of traditional TSL were generally composed of dipalmitoylphosphatidylcholine (DPPC), a saturated 16-carbon chain fatty acid with transition temperature (Tc) around 41 °C (32, 34 and 35). Increased drug release in response to heat was observed with pure DPPC TSL, however the amount and rate of release were relatively limited (33).

Adding either distearoyl phosphocholine (DSPC) or hydrogenated soy phosphocholine (HSPC) to the DPPC TSL increased packing incompatibility, bilayer permeability, and the amount and rate of cargo released (32, 36). 

Following the development of stealth PEGylated liposomes in 1990, PEGylated TSL were studied in 1994 (37). Koning and his coworkers investigated the optimum 1,2-distearoyl-sn-glycero-3-phosphoethanolamine-N-PEG(2000) (DSPE-PEG) percentage in TSL to achieve stealth liposomes with enhanced content release under mild HT. Different percentages of DSPE-PEG (1 to 10 mol %) were incorporated in TSL and cargo release as well as vesicle stability were monitored. Cargo leakage at physiological condition was reported with 6 mol% and higher DSPE-PEG. TSL with 5 mol% DSPE-PEG were stable at 37 °C, released 60% carboxyfluorescein in 1 min and almost 100% of cargo in 1 h at 42 °C and reported as the optimum TSL (38).

Dewhirst and Needham proposed the idea of formulating lysolipid based TSL (LTSL) that promoted rapid drug release in mild HT condition (39-42 °C). This formulation, composed of DPPC, 1-stearoyl-2-hydroxy-sn-glycero-3-phosphatidylcholine (MSPC), and DSPE-PEG (at 86:10:4 molar ratio), has been commercialized by Celsion under the trade name of ThermoDox^®^, the first and only TSL reaching clinical development. To ensure a sharp transition temperature, cholesterol was not added to the formulation; however, ThermoDox^® ^has a relatively short plasma residence time (39, 40).

Banno *et al. *found out the lysolipid (MSPC, 0-10 mol%) had a concentration-dependent effect on *In-vitro* drug release at 42 °C. However, within 1 h postinjection of LTSL, approximately 70% of lysolipid was lost (41).

There are two proposed mechanisms for cargo release upon HT with LTSL: i) formation of nanopores by lysolipids and DSPE-PEG in the bilayer during the phase transition (39) and ii) desorption of lysolipids from the bilayer membrane at T_c_ and formation of molecular scale defects for drugs escape (41). Lysolipids transfer from LTSL into biological membranes and their dissociation upon LTSL dilution in the blood stream may result in a negative impact on the temperature-sensitivity, premature drug release at physiological conditions, and its associated adverse effects (32). 

The HEAT trial evaluating the combination of ThermoDox^®^ and radiofrequency ablation in comparison to radiofrequency ablation alone for inoperable hepatocellular carcinoma treatment failed to reach its primary endpoint in progression-free survival. Allen and her coworkers extensively reviewed the possible reasons and factors underlying this failure (42). 

Lindner‘s group described a TSL based formulation based on 1,2-dipalmitoyl-sn-glycero-3-phosphodiglycerol (DPPG2) for doxorubicin (DOX) (43) and pyrimidine analogue gemcitabine (44). While similar release profile with ThermoDox^®^ was obtained (43), significantly prolonged plasma residence time was achieved (*i.e.* half-life of 5 h in rats and 9.6 h in hamsters) (45). Gemcitabine prodrug loaded DPPG2 TSL were also stable at 37 °C in serum and exhibited a temperature dependent cargo release > 40 °C. Plasma half-life of gemcitabine was significantly increased from 0.07 h (free drug) to 0.53-2.59 h (liposomal formulations). Therapy of soft tissue sarcoma BN175 with combination of gemcitabine loaded DPPG2 TSL and HT was the most effective treatment strategy (44).

Another approach for heat sensitizing of liposomal nanocarriers is to add synthetic polymers that are bilayer membrane disruptive in response to HT into lipid composition. Such polymers can either add to TSL to improve their heat-responsive functionality or incorporate to non-thermosensitive liposomes to make them thermosensitive. Temperature-sensitive polymers such as poly(N-isopropylacrylamide) (p(NIPAAm)) (46, 47), poly(N-vinyl ethers) (48, 49), elastin-like polypeptide (50), and poloxamers (Pluronic^®^) (51, 52), are among the most extensively studied temperature-sensitive polymer incorporated TSL.

Most of heat-responsive nanoparticles were not decorated with ligands for receptor-mediated targeting. However, a few recent studies have focused on design of next generation of TSL that combine targeting and triggered drug release. Various ligands such as folate (53, 54), trastuzumab antibody (herceptin) (49, 55 and 56), epidermal growth factor receptor (EGFR) specific Fab̕ fragment (57), affibodies (58), and peptides (57, 59 and 60) were conjugated to TSL.

**Figure 3:**
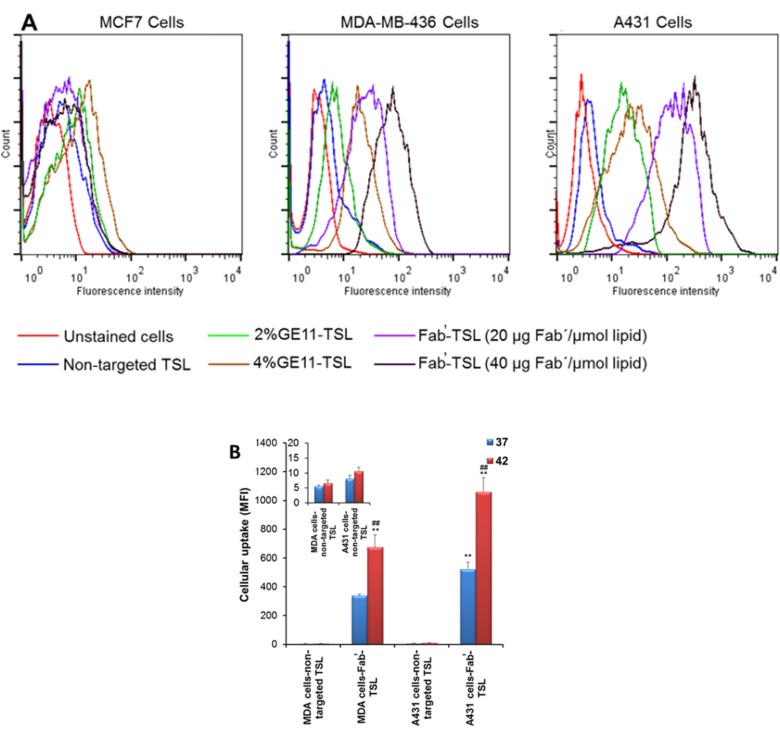
EGFR targeted thermosensitive liposomes (TSL) were successfully prepared for simultaneous tumor targeted and stimulus-responsive drug delivery. (A) Cellular uptake of labeled non-targeted and targeted TSL by cancer cells with different expression of EGFR receptors. Fab̕ modified TSL can more efficiently bind to the EGFR overexpressed cells as compared to GE11 decorated TSL. (B) Upon internalization dramatic intracellular cargo release was observed upon hyperthermia as confirmed by flow cytometry analysis. Reprinted from reference (57), Copyright 2016, with permission from Elsevier

Multifunctional TSL conjugated with anti-EGFR ligands (GE11 peptide and Fab̕ fragments of cetuximab) for targeted delivery and localized HT triggered release of chemotherapy were designed by our team (57) (Figure 3). Ligand decoration did not significantly alter the physicochemical characteristics of TSL. Compared to GE11 conjugated TSL, Fab̕-coated TSL (Fab̕-TSL) bound to the EGFR overexpressed cancer cells more specifically and efficiently as shown by flow cytometry and live cell imaging analyses. Calcein loaded Fab̕-TSL exhibited adequate stability at the physiological condition (<4% calcein released after 1 h at 37 °C in serum) and a temperature dependent release at > 40 °C. Combination of HT and Fab̕ modification enhanced cytotoxicity of DOX encapsulated TSL. The drug loaded Fab̕-TSL cytotoxicity was also correlated to EGFR density on the cancer cells (Figure 3) (57).

*Magnetic field-sensitive liposomes*

Magnetic nanoparticles, especially iron oxide nanoparticles, show great promise in biomedical applications due to their biocompatibility and unique features. They are used for magnetic resonance imaging (MRI), gene transfection, heat generation (or HT) under an alternating current (AC) magnetic field, targeted drug delivery, cell sorting, and cancer treatments (78-80).

The combination of magnetic nanoparticles and liposomes, commonly called “magnetoliposomes”, was first introduced in 1988 by De Cuyper and Joniau (81). Since then, magnetoliposomes have been used in MRI imaging, targeted drug delivery, and HT-mediated controlled drug release (82, 83).

Both bottom up and top down methods have been used for preparation of iron oxide nanoparticles. However, top down methods have some limitations on particle size and scale of production, thus bottom up techniques such as coprecipitation and thermal decomposition are more frequently used to prepare nanoparticles with hydrophilic and hydrophobic surfaces (84, 85). 

Magnetoliposomes can be formed by three different approaches: i) encapsulation of hydrophilic nanoparticle in the aqueous core of liposomes; ii) incorporation of hydrophobic nanoparticle within lipidic bilayer of liposomes; iii) binding magnetic nanoparticles on the surface of liposomes (80, 86). The first two methods are more commonly used.

Magnetic nanoparticles are one of the promising carriers for targeted delivery. By applying external field gradient from magnet, magnetic nanoparticles are attracted to the magnetic force. One of the main applications of magnetoliposomes is heat triggered cargo release mediated by an externally applied AC magnetic field at specific region. The heating of the magnetic nanoparticles depends on magnetic property of nanoparticles, frequency and amplitude of the magnetic field, and surrounding environment. Therefore, in order to control the temperature rise of magnetic nanoparticles, in depth optimization of magnetic field condition and surrounding environment is crucial for each magnetic nanocarrier (80). HT generated by magnetic nanoparticle under AC magnetic field can result in phase transition of the lipid bilayer from gel to liquid and trigger and control release of drugs encapsulated in magnetoliposomes. By tuning the bilayer composition of liposomes, the membrane phase transition can be adjusted to be around HT temperature (34, 82). Table 2 summarizes some recent researches of HT-mediated triggered release from magnetoliposomes. As an example of these researches, docetaxel loaded magnetoliposomes were prepared from purified magnetite and liposomes for gastric cancers. Tumor volume on 7^th^ day after treatment was at least 6 times lower in the animal group received docetaxel loaded magnetoliposomes in addition to applying magnetic field compared to free drug group. However, drug loaded liposomes treatment group without exposing to AC magnetic field showed comparable efficacy to drug solution group (87).

**Figure 4:**
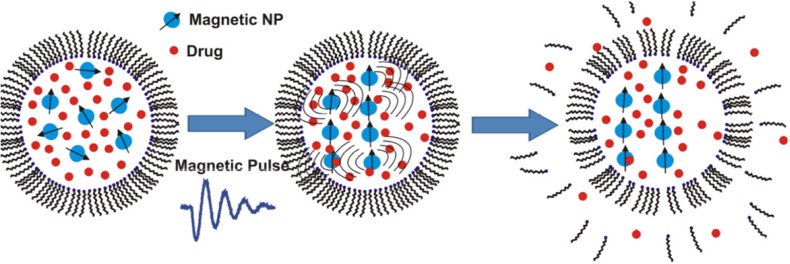
Fast release of the liposomes’ payload by using short magnetic pulses to disrupt the lipid bilayer of liposomes loaded with magnetic nanoparticles. Reprinted from reference (88) Copyright 2014, with permission from American Chemical Society

Stimulating drug release from magnetoliposomes by pulsed or low frequency magnetic field is a recent area of research that draws a lot of attention for triggering cargo release under controlled temperature (Table 2). For achieving fast drug release, short magnetic pulses were applied to disrupt the membrane of magnetoliposomes (Figure 4). Ultrasound generation under this magnetic field may also play a role in the drug release from the magnetic liposomes (88). Carboxymethyl dextran-coated magnetoliposomes with high loading ability for DOX were prepared and showed on-demand drug release under low-frequency alternating magnetic field. The hybrid nanostructures were demonstrated as a potential T2-weighted contrast agent for *In-vitro* MRI measurements (89).

Ligand conjugated magnetoliposomes have been also studied to achieve more specific drug delivery. Magnetoliposomes have been decorated with folate (90), hyaluronic acid (91, 92), anti-αvβ3 antibody (93), sugar moieties (94), and cell-penetrating peptides (CPPs) (95) to achieve ligand targeted magnetic liposomes. Hyaluronic acid, specifically bind to the CD44, is a promising ligand for tumor targeting due to overexpression of CD44 on various tumors including the colon, pancreas, breast, and ovarian (96). Hyaluronic acid decorated magnetoliposomes have been investigated for triggered release and targeted delivery of an anticancer drug (docetaxel). Docetaxel was incorporated in the vesicle bilayers while citric acid-coated magnetic nanoparticles were encapsulated in the aqueous cores. Targeted nanoparticles were about 190 nm and spherical in shape. Drug loaded targeted magnetoliposomes showed accelerated drug release under near-infrared laser irradiation and superior cellular uptake in comparison with the conventional non-targeted liposomes (91).

CPPs are attractive ligands in targeted drug delivery with the ability to transport various small and bulk cargoes intracellularly (97). However, their lack of specificity is a major limitation for CPPs’ systemic application (98). In order to control their biodistribution and present CPPs at target site, in a recent study, Lin *et al. *reported multifunctional targeted magnetoliposomes that encapsulated these ligands (95). CPP derived from penetratin was conjugated to DOX. CPP-DOX conjugate and Fe_3_O_4_ were co-encapsulated into lysolipid based TSL composed of DPPC:MSPC:DSPE-PEG2000 (87:3:10 mass ratio). The results demonstrated that the vesicles possessed appropriate size (98 nm) and encapsulation efficiency (87%). When AC magnetic field was applied for 30 min, about 86% CPP-DOX was released from the liposomes (12-fold compared to control condition). *In-vitro* cytotoxicity studies showed both CPP conjugation as well as HT-mediated by AC magnetic field improved anticancer efficacy. Moreover, *in-vivo* study in a breast xenograft model showed superior antitumor efficacy of multifunctional magnetoliposomes activated by AC magnetic field (95).

*Light-sensitive liposomes*

The success of light-triggered delivery system is dependent on adequate light source selection that can penetrate the tissues, photosensitizing properties of the therapeutic agents, and instrumentation. The preferred wavelengths are in the near-infrared (NIR) regions (~700 nm to 1100 nm) as the light penetration is more than 1 cm depth in the body (111, 112). Light absorbing pharmaceutical agents typically called as photosensitizers are promising candidates for photodynamic therapy (PDT). PDT is a minimally invasive cancer treatment generally based on light-mediated excitation of a photosensitizer resulting in localized production of reactive oxygen species (ROS) and destruction of nearby unwanted biological agents (113, 114). Due to hydrophobic properties and non-specific biodistribution of photosensitizers, their application in cancer therapy meets technical challenges. Nanoparticles especially liposomal formulations of photosensitizers are attractive systems for improved and targeted delivery of photosensitizers (114, 115) (Table 3). In formulation of successful photo-triggerable liposomes, retention of vesicle stability and entrapped cargo before accumulation at the target site as well as efficient activation/destabilization of liposomes in the tissue by the source light are very important parameters (111, 112). Visudyne is a successful example of photosensitizer (verteporfin) liposomal formulations that is currently clinically used. 

A number of photo-triggerable synthetic phospholipids have been investigated to undergo various chemical processes including photosensitization, photopolymerization, photooxidation, photoisomerization, or the degradation of photocleavable lipids that have been explained in recent review articles (116, 117). The majority of these nanosystems were based on light-triggered modifications in conjunction with a photosensitizing molecule either incorporated in the bilayer membrane or encapsulated in the aqueous core. The photo-induced modifications were mostly irreversible changes with the exception of phospholipid molecules with photo-triggering capability via the cis–trans isomerization (111, 112 and 117). 

**Figure 5:**
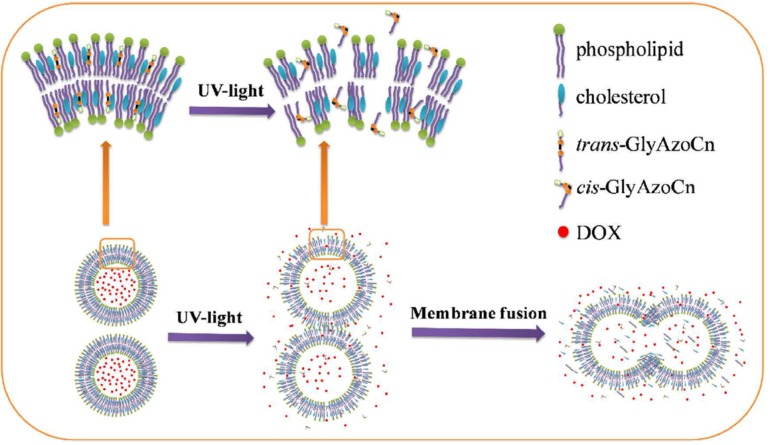
The diagram of photoisomerization induced burst release of doxorubicin from liposomes embedded by azobenzene-contained glycolipid. Reprinted from reference (120) Copyright 2017, with permission from American Chemical Society

Azobenzenes are a class of chemical compounds that undergo photoisomerization of their cis and trans isomers. Bis-Azo PC (a photochromic lipid) and azobenzene cholesterol derivatives were studied (118, 119). Recently, newly structured azobenzene derivatives, azobenzene-contained glycolipids, have been synthesized. The photo-induced control of DOX release from liposomes was investigated (Figure 5). The isomerization process in ethanol solution was much faster than that in the liposome bilayer, indicating the hindering effect of surrounding lipids in the liposomal bilayer. Among the synthesized azobenzene-contained glycolipids, GlyAzoC7 was shown to be the most favorable photosensitive actuator for controlling cargo release. In the dark, less than 10% drug leakage was observed in 10 h but nearly 100% of cargoes instantaneously released with ultraviolet (UV) irradiation (120). However, due to limited tissue penetration of UV/visible wavelengths, achieving a suitable photo-triggering at *in-vivo* conditions was a challenging issue. A new hybrid vesicle based on azobenzene liposome and phosphatidylcholine modified upconversion nanoparticle (UCNP) was designed for precise remote control of drug release using NIR light. The encapsulated UCNPs converted NIR light into the UV/visible region emissions which can be immediately absorbed by the photoresponsive azobenzene amphiphilic molecules in the liposomal bilayer (121).

Photocleavable liposome is another approach to trigger drug release in response to light and designed on the basis of destabilization and disruption of liposome membrane by breakdown products of irradiation. Chandra and co-workers synthesized several amphiphiles containing a nitrobenzyl moiety separating a polar amino acid headgroup from a long hydrophobic tail (122, 123). To develop analogues with closer structure to lipids, a photocleavable 2-nitrobenzyl group embedded within the acyl chain was synthesized by Bayer *et al. *(124). 

Another research groups have focused on photopolymerization rendering the destabilization of liposomal bilayer by intermolecular photo-crosslinking of phospholipids. bis-sorbyl phosphatidylcholine and 1,2-bis(tricosa-10,12-diynoyl)-sn-glycero-3-phosphocholine (DC_8,9_PC) are some examples of photopolymerizable lipids that have been studied. Puri and her coworkers developed DPPC:DC_8,9_PC formulations and the cargo release (DOX or calcein) occurred upon treatment with a 514 nm laser. Photo-triggering occurred primarily via a type-I photoreaction process (125, 126).

In addition to light-sensitive liposomes, photo-stabilized liposomes are attractive candidates for sustained drug delivery. In this approach, a photopolymerizable group was introduced into lipid bilayer to prepare plasma stable liposomes. An example of photo-stabilized liposomes was vesicles prepared from the polymerizable lipid, 1,2-dipalmitoyl-sn-glycero-3-phospho-N-(2-hydroxymethyl)-3,5-divinylbenzamide (DPPE-DVBA), that have been demonstrated to photo-crosslink in the presence of UV light (127).

*Ultrasound-sensitive liposomes*

Ultrasound offers an easy and non-invasive method for precise drug delivery because its energy can disrupt nanostructures that stably encapsulated cargoes before triggering. Furthermore, enhancement of drug transport across cell membranes and a synergistic effect between the pharmacological activity of some drugs and ultrasound effects were observed. In ultrasound-mediated triggered drug carriers, acoustic parameters should be carefully tuned to be energetic enough to actuate drug release while avoiding harmful damage to cells and tissues (137, 138). Compared to high-frequency ultrasound (1–3 MHz), low frequency ultrasound (20–100 kHz) can be a more effective trigger for drug release and penetrate deeper into tissues. However, it does not allow for sharp focusing (139). In this part, non-thermal effects of ultrasound to enhance drug release from liposomal carrier are discussed and examples of recent studies are presented in Table 4.

Liposomes are rather transparent to ultrasound; however, need to contain a gas phase for being sensitive to ultrasound. Therefore, acoustically active liposomes (echogenic liposomes and bubble liposomes) that contain a gas phase are designed in order to respond to ultrasound. To prepare acoustically triggered liposomes, different strategies including an internal gas bubble, a liquid phase changeable to a gas bubble upon insonation, bubbles attached to the vesicle exterior parts, and bubbles reside in the close proximity of liposomes have been used. Two main mechanisms have been proposed for shear stress and cavitation of ultrasound to trigger vesicle content release: i) producing small pores for enhanced permeability or ii) destabilization and disruption of the entire liposome. 

In the first strategy to prepare ultrasound-responsive liposomes, gas bubbles (generally micron-sized) are nested inside liposomal vesicles. These delivery systems can be prepared by either mixing and sonication of gas bubbles with phospholipid mixture (140), or sonication of liposomes in the presence of perfluorocarbon gas (141), or gas generation inside the liposomes by a chemical reaction (*e.g.* bicarbonate solution) (142).

Suzuki *et al. *reported effective gene delivery system by using the bubble liposome and sonoporation for IL-12 corded plasmid DNA delivery (143). This approach dramatically suppressed tumor growth and the therapeutic effect was T-cell dependent (143). One advantage of bubble liposomes over echogenic liposomes is that the bubble liposomes are smaller in size with average diameter generally less than 500 nm, compared to micron sizes for echogenic liposomes (143, 144).

**Figure 6:**
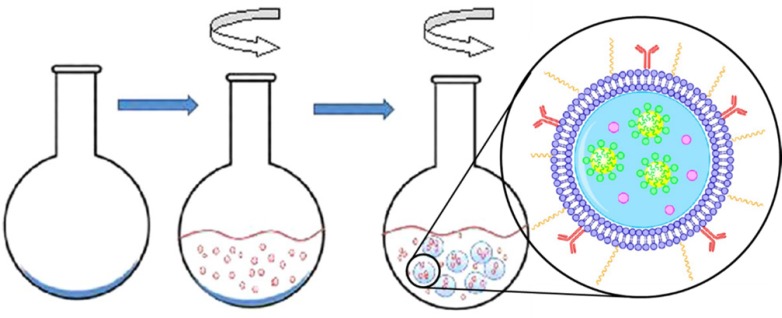
One-step process of eLiposomes production. Phospholipid is deposited on the flask and an emulsion is added. eLiposomes form while the flask is rotated. Reprinted with minor modification from reference (145), with permission from American Chemical Society, Copyright 2012

To achieve nanosized vesicles for extravasation or endocytosis, another strategy has been invented that the gas bubbles are not pre-existing, but rather form upon the ultrasound triggering event. This approach allows vesicles with diameter less than 500 nm. Pitt and his coworkers developed an emulsion-containing liposome (eLiposome). The ultrasound application can cause changing the emulsion droplet to gas, thus increasing the volume inside the liposomes and leading to vesicle rupture and the cargo release (145-147). Two methods were proposed for eLiposome preparation. In the first method, emulsion droplets were made of perfluorohexane or perfluoropentane and stabilized with phospholipids. A thin layer of phospholipids was dried in a round-bottomed flask. The emulsion was added to the flask and hydrated the phospholipids forming liposomes around the emulsions (one-step preparation, Figure 6). In the second method, liposomes and emulsions were made separately, and then mixed using ultrasound (145).

The third category of ultrasound-sensitive liposomes is designed by small liposomes that are attached to larger gas bubbles. The liposomes are usually around 100 nm and loaded with the therapeutic agents while the bubbles are ~1–3 μm and contain a perfluorocarbon gas (148, 149). When exposed to ultrasound the bubble cavitates violently, and the resulting shock waves and shear forces disrupt the nearby vesicle bilayer and release its content. As an example of these studies, Cool *et al. *prepared microbubbles with drug containing liposomes at their surface in one single step. Liposomes prepared from DPPC, cholesterol, and DSPE-PEG and loaded with indocyanine green (ICG) as a model drug. Mirobubbles were composed of the perfluorobutane gas. This strategy led to enhanced liposome extravasation (148). 

Some studies have described ligand targeted ultrasound-responsive liposomes by using antibodies (150), folate (151), and CPPs (152).

*Internal stimuli-responsive liposomes*

*pH-sensitive liposomes*

pH-sensitive liposomes have been designed to trigger and promote efficient release of entrapped cargoes in response to an acidic environment. The pH of blood and extracellular fluid of normal tissues is approximately 7.4 whereas in extracellular microenvironment of tumor, pH is between 6.0 and 7.0 (160, 161). The acidosis in tumor tissue can be explained by the poor organization and dysfunctional vasculature, heterogeneous blood flow, and insufficient nutrient delivery. This condition ultimately forces cells to generate energy from anaerobic glycolytic metabolism of glucose to lactic acid. Limited clearance and increased accumulation of lactic acid due to reduced blood flow lead to a pH reduction in the tumor microenvironment, as mentioned above (162). However, the pH value of the tumor interstitial fluid rarely declines below 6.5, thus designing liposomal carrier to disrupt in response to such a narrow pH change is technically challenging (161). On the other hand, following binding to cancer cells, the vesicles can be up-taken and internalized through endocytosis and retained in endosomal and lysosomal compartments. The promising potential of pH-sensitive liposomes lies in their ability in fusion or destabilization after cell internalization at the endosomal stage with pH values in range of 4.5 – 5.5 (mainly due to the activity of vacuolar-type proton ATPase) (163), thereby preventing their contents degradation at the lysosomal level and promoting cargo release into the cytoplasm. This process known as ‘endosomal escape’ results in releasing drug payload into the cytosol and also far from the transmembrane efflux pumps, thereby at least partly preventing lysosomal degradation and circumventing drug resistance development in tumor cells (164, 165). To date, various liposomal carriers have been designed to respond to either low extracellular pH in tumors or endosomal pH compartments (Table 5).

Dioleoylphosphatidylethanolamine (DOPE) is the most commonly used pH-sensitive lipid. DOPE has a cone shape due to small and minimally hydrated polar head group that occupies a lower volume compared with its acyl chains. The inverted cone shape of DOPE lipid has a tendency to form an inverted hexagonal H_II_ shape at physiological pH because of strong interactions between the phosphate and amine groups of the polar head groups. DOPE by itself with these structural aspects cannot form lipid bilayers at neutral pH. DOPE combined with amphiphilic molecules containing a protonatable acidic group, such as cholesteryl hemisuccinate (CHEMS) and oleic acid have been used to prepare pH-sensitive liposomes. The electrostatic repulsion between deprotonated carboxylate and phosphate groups allows the formation of bilayer structures at neutral pH. At acidic pH, destabilization of liposomes is mediated by the protonation of carboxylate groups, suppressing charge repulsion in the bilayer, and consequently resulting in the reversion of DOPE molecules into their inverted hexagonal phase (161, 166 and 167).

Besides a considerable number of researches on pH-sensitive liposomes prepared from DOPE derivatives, a few studies have recently described incorporation of novel pH-sensitive lipids. Szoka and his coworkers synthetized a novel acid-labile lipid containing a linear ortho ester linker between cholesterol-derived lipid tail and its dimethylethanolamine-type cationic head group. Liposomes, composed of this acid-labile lipid and DOPE, were used for gene delivery. Compared to the acid-stable control, pH-sensitive lipoplexes increased the luciferase gene expression by 5- to 10-fold both in CV-1 cells (a monkey fibroblast cell line) and following intratracheal administration in CD-1 mice (168). Harashima group introduced another cationic pH-sensitive lipid, YSK05 (a tertiary amine containing lipid with structure similar to DOTAP) for improving the delivery of liposomal siRNA and gene silencing (169, 170).

The other approach to prepare pH-sensitive liposomes is by incorporation of pH-sensitive fusogenic peptides either derived from viruses (like haemagglutinin, gp41, and diINF-7), bacteria (such as listeriolysin O and diphtheria toxin), and plants (*e.g.* ricin, saporin, and gelonin) or synthetic materials (*e.g.* GALA, KALA, and surfactants) (for review see (171)).

Liposomes enriching with pH-sensitive polymers such as *N*-isopropylacrylamide (NIPAM) (172, 173), poly(glycidol)s (174, 175), and poly(alkyl acrylic acid)s (176, 177) have also proposed for acid-responsive delivery. Simplicity of preparation and lower immunogenicity are probably two main advantages of these carriers to peptidic pH sensitizers. As an example of these studies, Yuba *et al. *(175) investigated the relationship between backbone structure of pH-sensitive poly(glycidol) derivatives and their interaction with the membrane. A stronger interaction with the membrane was observed with hyperbranched poly(glycidol) derivatives than the linear polymers. Increasing degree of polymerization of hyperbranched poly(glycidol) derivatives enhanced their bilayer interaction as well. Liposomes modified with these polymers effectively delivered their contents into the cytosol of dendritic cells (175).

In spite of the success of ‘stealth’ nanosystems (PEGylated pH-sensitive liposomes) in achieving stability and long-circulation, DSPE-PEG containing pH-sensitive liposomes showed reduced pH-sensitivity and cellular uptake. The reduced ability of PEGylated shell to come into close proximity of cancer cells and endosomal membranes is one of main explanations for reduced internalization and pH-responsiveness. Aiming to overcome the instability of non-PEGylated liposomes in addition to maintain adequate pH sensitivity, some studies have been focused on designing pH-sensitive liposomes with a cleavable PEG chain (178-181). 

**Figure 7:**
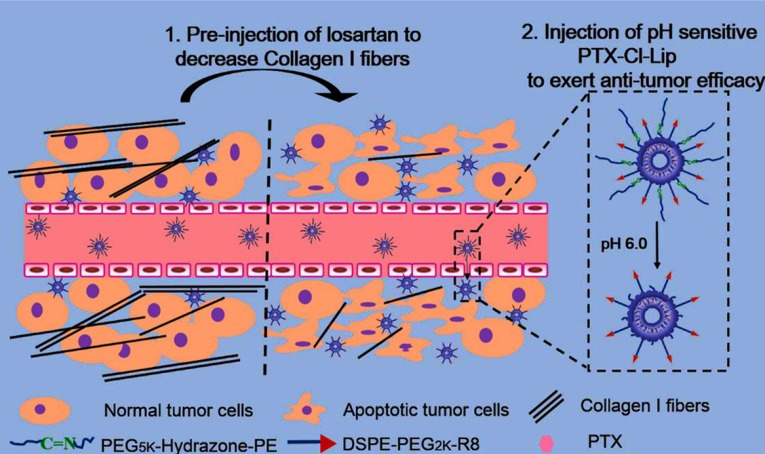
CPP decorated pH-sensitive cleavable liposomes are designed and loaded with paclitaxel. The CPP (R8 peptide) is conjugated to a short PEG and long PEG chains are linked to liposomal surface by acid-sensitive hydrazone bond. Before liposomes injection, free losartan is administered to deplete the collagen I and facilitate liposomes deep penetration into tumors. Low extracellular pH in the cancer cell proximity causes long PEG detachment and exposes CPP to the tumor cell. Reprinted from reference (178) Copyright 2015, with permission from American Chemical Society

In a recent study, CPP decorated pH-sensitive cleavable liposomes were designed and loaded with paclitaxel. The CPP (R8 peptide) was conjugated to a short PEG group and long PEG chains were linked to liposomal surface by hydrazone bond. Before liposomes injection, free losartan was administered to deplete the collagen I and facilitated liposomes deep penetration into tumors. After taking the advantage of increasing plasma residence time and passive targeting by the long PEG, low extracellular pH in the cancer cell proximity caused long PEG detachment and exposed CPP to the tumor cell. This approach can overcome limited uptake of PEGylated liposomes as well as non-specificity of R8 peptide (Figure 7) (178).

**Figure 8.**
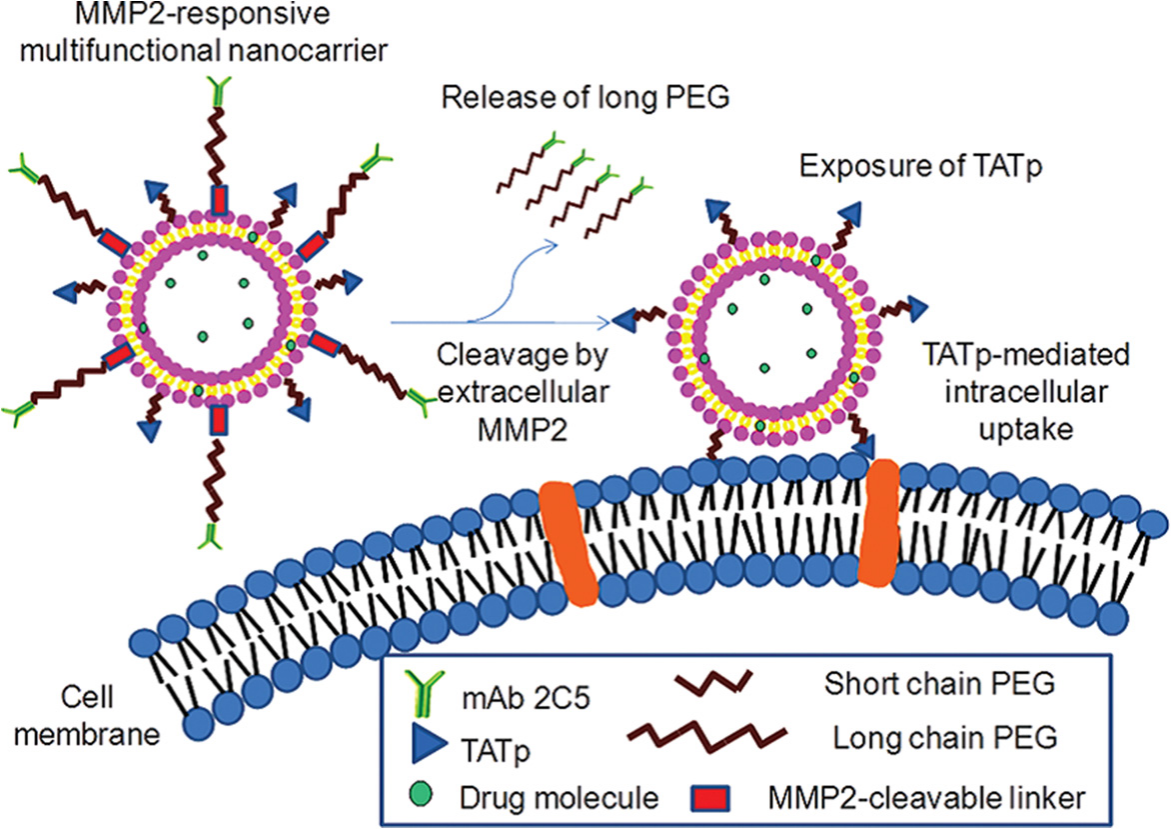
A dual antinucleosome monoclonal antibody and TAT peptide targeted MMP-2-responsive multifunctional liposomal delivery system is designed. Upon nanocarrier accumulation in tumors and specific targeting of cancer cells, in response to up-regulated extracellular MMP-2 in tumors, the hidden surface-attached TAT peptides expose and enhance cellular internalization of liposomes. Reprinted from reference (215) Copyright 2012, with permission from American Chemical Society

**Table 1.** Examples of recent studies on thermosensitive liposomes

Stage of study	Cargo	*In-vivo *tumor model	Components	Targeting ligand	Reference
***In-vitro***	Marker	_	DPPC, DSPC, DPPG2	_	(61)
***In-vitro***	Arsenic trioxide	_	DPPC, MPPC	_	(62)
***In-vitro***	DOX	_	DPPC, HSPC, Chol, DSPE-PEG, p(NIPAAm-*co*-PAA)	_	(63)
***In-vitro***	_	_	DPPC, C12H25-PNIPAM-COOH, PnBA-PNIPAM	_	(64)
***In-vitro***	DOX, Marker	_	DPPC, Chol, Dimyristoylphosphatidic acid, 2C12-p(NIPMAM-co-NIPAM)	_	(65)
***In-vitro***	DOX	_	DPPC, DSPC, DSPE-PEG, short-chain glucosylceramide,	_	(66)
***In-vitro*** **/** ***In-vivo***	DOX, Marker	Murine sarcoma (BFS-1 cells)	DPPC, DSPC, DSPE-PEG	_	(67)
***In-vitro*** **/** ***In-vivo***	Cisplatin, Marker	Cervical carcinoma (ME-180 cells)	DPPC, DPPG, MSPC, DSPE-PEG	_	(68)
***In-vitro*** **/** ***In-vivo***	DOX, Marker	Breast cancer (MDA-MB-435 cells)	DPPC, Chol, DSPE-PEG, ammonium bicarbonate, gold nanorods	_	(69)
***In-vitro*** **/** ***In-vivo***	5-FU, Marker	Colorectal adenocarcinoma (HT-29 cells)	DPPC, Chol, DSPE-PEG	_	(70)
***In-vitro*** **/** ***In-vivo***	Cisplatin, Marker	Cervical carcinoma (ME-180 cells)	DPPC, DPPG, MSPC, DSPE-PEG,	_	(71)
***In-vitro*** **/** ***In-vivo***	Marker	Murine melanoma (B16B16 cells)	DPPC, DSPC, DSPE-PEG, DPTAP	_	(72)
***In-vitro*** **/** ***In-vivo***	DOX	Melanoma (BLM cells)	DPPC, DSPC, DSPE-PEG	_	(17)
***In-vitro*** **/** ***In-vivo***	DOX	Murine squamous cell carcinoma (SSC-7 cells)	DPPC, Chol, DSPE-PEG, Elastin-like polypeptide	_	(73)
***In-vitro*** **/** ***In-vivo***	DOX, Marker	Murine mammary tumor (EMT-6 cells)	DPPC, Chol, DSPE-PEG, Elastin-like polypeptide	_	(50)
***In-vitro*** **/** ***In-vivo***	Oxaliplatin	Lewis Lung Cancer Cell (LLCC)	DPPC, MSPC, DSPE-PEG, Poloxamer 188	_	(52)
***In-vitro*** **/** ***In-vivo***	DOX, Marker	_	DPPC, Polxamer 188	_	(51)
***In-vitro*** **/** ***In-vivo***	DOX, Marker	Murine colon cancer (C26 carcinoma cells)	EPC, DOPE, Chol, DSPE-PEG, EOEOVE-ODVE	_	(74)
***In-vitro***	DOX	_	DPPC, Chol, Brij78	Pamidronate	(75)
***In-vitro***	DOX, Marker	_	DPPC, DSPC, DSPE-PEG	Cetuximab (Fab' fragments), GE11 peptide	(57)
***In-vitro*** **/** ***In-vivo***	DOX, Marker	Breast cancer (MCF-7 cells)	DPPC, DSPC, Chol, DSPE-PEG, Elastin-like polypeptide	cRGD peptide	(50)
***In-vitro*** **/** ***In-vivo***	DOX, Marker	Murine melanoma (B16B16 cells)	DPPC, DSPC, DSPE-PEG	cRGD peptide	(60)
***In-vitro*** **/** ***In-vivo***	DOX, Marker	Multi resistant breast cancer (MCF-7/ADR cells)	DPPC, MSPC, DSPE-PEG	CREKA peptide	(76)
***In-vitro*** **/** ***In-vivo***	DOX, Marker	Epidermoid carcinoma (KB cells)	DPPC, Chol, DSPE-PEG	Folate	(53)
***In-vitro*** **/** ***In-vivo***	DOX, Marker	Ovarian carcinoma (SK-OV3 cells)	EPC, Chol, DSPE-PEG_5000_, EOEOVE-ODVE	Trastuzumab	(49)
***In-vitro*** **/** ***In-vivo***	siRNA-CPP, Marker	Fibrosarcoma (HT1080 cells)	DPPC, MSPC, DSPE-PEG	NGR peptide	(77)

**Abbreviations: **Brij78: Polyoxyethylene stearyl ether; C12H25-PNIPAM-COOH: C12H25-poly(N-isopropylacrylamide)-COOH; 2C12 (PNIPMAM co NIPAM): 2C12 (poly N-isopropylmethacrylamide co poly N-isopropylacrylamide); Chol: Cholesterol; CREKA peptide: Cys-Arg-Glu-Lys-Ala; cRGD peptide: Cyclic Arg-Gly-Asp peptide; DOPE: 1,2-Dioleoylsn-glycero-3-phosphoethanolamine; DOX: Doxorubicin; DPPC: 1,2-Dipalmitoyl-sn-glycero-3-phosphocholine; DPPG: 1,2-Dipalmitoyl-snglycero-3-phosphoglycerol; DPTAP: 1,2-Dipalmitoyl-3-trimethylammonium-propane; DSPC: 1,2-Distearoyl-sn-glycero-3-phosphocholine; DSPE-PEG: 1,2-Distearoyl-sn-glycero-3-phosphoethanolamine-(polyethylene glycol)-2000; EOEOVE-ODVE: Block copolymer compounds of octadecyl vinyl ether and 2-(2-ethoxy)ethoxyethyl vinyl ether; EPC: Egg phosphatidylcholine; 5-FU: 5-Fluorouracil; HSPC: Hydrogenated soy phosphatidylcholine; MPPC: Monopalmitoylphosphatidylcholine; MSPC: Mono-stearoyl-sn-glycero-3-phosphatidylcholine; NGR peptide: Asn-Gly-Arg peptide; PnBA-PNIPAM: Poly(n-butylacrylate-b-N-isoropylacrylamide)-poly(N-isopropylacrylamide); p(NIPAAm-co-PAA): Copolymer of N-isopropylacrylamide conjugated with propylacrilic acid; siRNA-CPPs: Small (or short) interfering RNA via disulfide-bonds conjugated with cell penetrating peptides

**Table 2:** Examples of recent studies on magnetic field-sensitive liposomes

Stage of study	Cargo	*In-vivo* tumor model	Components	Magnetic field specifications^1^	Reference
***In-vitro***	5-FU	_	PC, Fe_3_O_4_ nanoparticles	f = 250 kHz, H = 4 kA/m	(99)
***In-vitro***	Gemcitabine	_	DPPC, Chol, Fe_3_O_4 _nanoparticles	f = 356 kHz, H = 30 kA/m, B = 2T	(100)
***In-vitro***	Paclitaxel	_	DPPC, PG, Fe_3_O_4 _nanoparticles	f = 423kHz, H = 10 kA/m	(101)
***In-vitro***	Marker	_	DSPC, POPC, SOPC, DSPE-PEG, Palmityl-nitroDOPA, Iron oxide nanoparticles	f = 230 kHz	(102)
***In-vitro***	Curcumin	_	DPPC, Chol, DSPE-PEG, Fe_3_O_4_ nanoparticles		(103)
***In-vitro*** **/** ***In-vivo***	Arsenic trioxide	Hepatocarcinoma (SMMC-7721 cells)	DPPC, Chol, Mn0.5Zn0.5Fe_2_O_4_ nanoparticles	f = 230 kHz	(104)
***In-vitro*** **/** ***In-vivo***	Docetaxel	Gastric cancer (MKN45 cells)	DLPC, DOPE, FeFe_2_O_4_ nanoparticles	f = 478 kHz, H = 6.36 kA/m, P = 1kW	(87)
***In-vitro*** **/** ***In-vivo***	DOX-CPP conjugate, Marker	Breast adenocarcinoma (MCF-7 cells)	DPPC, MSPC, Fe_3_O_4_ nanoparticles	f = 423kHz, H = 10 k A/m	(95)
***In-vitro***	Marker	_	SPC, Fe_3_O_4_ nanoparticles	f = 20 kHz, H = 100 A/m	(105)
***In-vitro***	Marker	_	HSPC, Chol, Fe_3_O_4_ nanoparticles	f = 20 kHz, H = 60 A/m	(106)
***In-vitro***	Marker	_	Cetyltrimethylammonium chloride, Myristic acid, Fe_3_O_4_ nanoparticles	f = 520 kHz, H = 28 kA/m, B = 145 mT	(107)
***In-vitro***	DOX	_	SPC, Fe_3_O_4_ nanoparticles	f = 50 Hz, B = 30 mT	(89)
***In-vitro***	Marker	_	DPPC, DSPC, Chol, Fe_3_O_4_ nanoparticles	f = 214.8 Hz, B = 3 T	(88)
***In-vitro*** **/** ***In-vivo***	Oxaliplatin, Gemcitabine	Breast cancer (MCF-7 cells)	PC, DMPG, Chol, Fe_3_O_4_ nanoparticles	B = 0.5 T	(108)
***In-vitro***	DOX, Marker	_	DPPC, Chol, DSPE-PEG, Fe_3_O_4_ nanoparticles	f = 290 kHz, H = 12 kA/m	(109)^2^
***In-vitro***	DOX	_	DOPC, DSPE-PEG, DPTAP, Fe_2_O_3_ nanoparticles	f = 287 kHz, H = 5.9 ×10^5^ kA/m, P = 1 kW	(90)^2^
***In-vitro*** **/** ***In-vivo***	Marker	Hepatocarcinoma (CD90+ stem cells)	DPPC, DSPE-PEG, Fe_3_O_4_ nanoparticles	f = 200 kHz	(110)^3^

**Abbreviations: **Chol: Cholesterol; CPP: Cell penetrating peptide; DLPC: Dilauroylphosphatidylcholine; DMPG: 1,2-Dimyristoyl-sn-glycero-3-phosphoglycerol; DOPC: 1,2-Dioleoyl-sn-glycero-3-phosphocholine; DOPE: 1,2-Dioleoylsn-glycero-3-phosphoethanolamine; DOX: Doxorubicin; DPPC: 1,2-Dipalmitoyl-sn-glycero-3-phosphocholine ; DPTAP: 1,2-Dipalmitoyl-3-trimethylammonium-propane; DSPC: 1,2-Distearoyl-sn-glycero-3-phosphocholine; DSPE-PEG: 1,2-Distearoyl-sn-glycero-3-phosphoethanolamine-(polyethylene glycol)-2000; 5-FU: 5-Fluorouracil; HSPC: Hydrogenated soy phosphatidylcholine; MSPC: Mono-stearoyl-sn-glycero-3-phosphatidylcholine; Palmityl-nitroDOPA: Palmityl-nitro 3,4-dihydroxyphenylalanine; PC: Phosphatidylcholine; PG: 1-Palmitoyl-2-oleoyl-sn-glycero-3-phospho-rac-glycerol; POPC: 1-Palmitoyl-2-oleoyl-sn-glycero-3-phosphocholine; SOPC: 1-Stearoyl-2-oleoyl-sn-glycero-3-phosphocholine; SPC: Soybean phosphatidylcholine

1 B, Magnetic induction; f, Frequency; H, Magnetic intensity; M, Magnetization; P, Magnetic power.

2 Folate decorated nanocarrier for targeted delivery.

3 CD90 antibody decorated nanocarrier for targeted delivery.

**Table 3:** Examples of recent studies on light-sensitive liposomes

Stage of study	Cargo	*In-vivo* tumor model	Components	Light wavelength	Photosensitizing agent or group	Reference
***In-vitro***	Marker	_	DSPC, DMPC, Chol	UV (365 nm)	ZnPC	(128)
***In-vitro***	Marker	_	DPPC, DSPC, Lyso PC, DSPE-PEG	NIR (808 nm)	ICG	(129)
***In-vitro***	Marker	_	SOPC, DOPC, SLPC	Visible (590 nm)	m-THPP, Pheophorbide a, Verteporfin	(130)
***In-vitro***	DOX, Marker	_	DPPC, DSPE-PEG, DC_8,9_PC	Visible (514 nm)	_	(125)
***In-vitro***	Marker	_	DPPC, MPPC, DPPE-PEG	NIR (760 nm)	Gold nanoparticles	(131)
***In-vitro***	Marker	_	DSPC, DPPC	UV (365 nm)	Gold nanoparticles (Au NPs)	(132)
***In-vitro***	Marker	_	EPC, DPPC, DOPC, DLiPC, DPhPC	Visible (532, 633 nm)	AlPcS3, ZnPcGlyc4, Chlorin e6	(133)
***In-vitro***	Marker	_	EPC, PVA carrying a malachite green moiety	UV	_	(134)
***In-vitro***	Marker	_	DPPC, DSPE-PEG, DC_8,9_PC	Visible (514 nm)	_	(126)
***In-vitro***	Marker	_	DSPC, photocleavable lipid	UV (365 nm)	Amphiphilic lipids containing amino acids and o-nitrobenzyl groups	(123)
***In-vitro***	DOX	_	HSPC, DMPG, Chol	UV (365 nm)	Azobenzene moiety	(120)
***In-vitro***	Marker	_	DMPC, DMPG, DMPE, DMPS,	UV (365 nm), Visible (532 nm)	Azobenzene moiety	(135)
***In-vitro*** **/** ***In-vivo***	DOX	Breast cancer (MCF 7 cells)	DSPC, DSPE-PEG	NIR (980 nm)	Upconversion nanoparticles; Azobenzene moiety	(121)^1^
***In-vitro*** **/** ***In-vivo***	DOX, Marker	Ovarian carcinoma (SKOV3 cells), Lung adenocarcinoma (A549 cells)	DPPC, HSPC, Chol, DSPE-PEG	NIR	Gold nanoparticles	(136)^2^

**Abbreviations: **AlPcs3: Aluminum trisulfophthalocyanine; Chol: Cholesterol; DC8,9PC: 1,2-Bis(tricosa-10,12-diynoyl)-sn-glycero-3-phosphocholine; DLiPC: Dilinoleoylphosphatidylcholine; DMPC: 1,2-Dimyristoyl-sn-glycero-3-phosphocholine; DMPE: Dimyristoyl phosphatidylethanolamine; DMPG: 1,2-Dimyristoyl-sn-glycero-3-phosphoglycerol; DMPS: Dimyristoyl phosphatidylserine; DOPC: 1,2-Dioleoyl-sn-glycero-3-phosphocholine; DOX: Doxorubicin; DPhPC: Diphytanoylphosphatidylcholine; DPPC: 1,2-Dipalmitoyl-sn-glycero-3-phosphocholine; DPPE-PEG: Dipalmitoylphosphatidylethanolamine-[N-methoxy(polyethylene glycol)-2000]; DSPE-PEG: 1,2-Distearoyl-sn-glycero-3-phosphoethanolamine-(polyethylene glycol)-2000; DSPC: 1,2-Distearoyl-sn-glycero-3-phosphocholine; EPC: Egg phosphatidylcholine; HSPC: Hydrogenated soy phosphatidylcholine; ICG: Indocyanine green; Lyso PC: 1-Stearoyl-2-hydroxy-sn-glycero-3-phosphocholine; MPPC: Monopalmitoylphosphatidylcholine; m-THPP: [5,10,15,20-tetrakis(3-hydroxyphenyl)porphyrin]; NIR: Near infrared; PC: Phosphatidylcholine; PVA: Poly(vinyl alcohol); SLPC: 1-Stearoyl-2-linoleoyl-sn-glycero-3-phosphocholine; SOPC: 1-Stearoyl-2-oleoyl-sn-glycero-3-phosphocholine; UV: Ultraviolet; ZnPC: Zinc phthalocyanine; ZnPcGlyc4: Neutral zinc phthalocyanine with four glycerols attached to the peripheral (4,5) positions of the isoindoline subunits

1 Folate decorated nanocarrier for targeted delivery.

2 HER2 antibody decorated nanocarrier for targeted delivery.

**Table 4:** Examples of recent studies on ultrasound-sensitive liposomes

Stage of study	Cargo	*In-vivo* tumor model	Components	Gas type	Ultrasound frequency	Reference
***In-vitro***	DOX, Marker	_	DSPC, Chol, DSPE-PEG, DOPE	_	40 kHz	(153, 154)
***In-vitro***	Marker	_	DSPC, Chol, DSPE-PEG, DOPE	_	1.13 MHz	(155)
***In-vitro***	DOX	_	DPPC, Chol, DSPE-PEG-SPDP	Decafluorobutane	1 MHz	(156)
***In-vitro***	Marker	_	POPC, Lipopeptide	_	3 MHz	(157)
***In-vitro***	Thrombin, Marker	_	PC, Chol, PEG150 stearate, Biotin-PEG3400-PC	Decafluorobutane	1 MHz	(149)
***In-vitro***	_	_	DPPC	Perfluorohexane	20 kHz	(146)
***In-vitro*** **/** ***In-vivo***	Marker	_	DPPC, Chol, DSPE-PEG, PDP	Decafluorobutane	1 MHz	(148)
***In-vitro*** **/** ***In-vivo***	Marker	Prostate tumor (22Rv1 cells)	DSPC, Chol, DSPE-PEG, DOPE	_	1.1 MHz	(158)
***In-vitro*** **/** ***In-vivo***	DOX, Marker	Metastatic murine melanoma (B16F10 luciferase cells)	HSPC, Chol, DSPE, DSPE-PEG	Sulphur hexafluoride	0.5 MHz	(159)
***In-vitro*** **/** ***In-vivo***	IL-12 corded pDNA	Murine ovarian carcinoma (OV-HM cells)	DSPC, DSPE-PEG	Perfluoropropane	1 MHz	(143)
***In-vitro***	Marker	_	DPPC, DSPC, DMPC, DSPE-PEG, DLPA, DPPA	Perfluorohexane, Perfluoropentane	20 kHz	(145)^1^

**Abbreviations:** Chol: Cholesterol; DLPA: 1,2-Didodecanoyl-snglycero-3-phosphate (sodium salt); DMPC: 1,2-Dimyristoyl-sn-glycero-3-phosphocholine; DOPE: 1,2-Dioleoylsn-glycero-3-phosphoethanolamine; DOX: Doxorubicin; DPPA: 1,2-Dipalmitoyl-sn-glycero-3-phosphate; DPPC: 1,2-Dipalmitoyl-sn-glycero-3-phosphocholine; DSPC: 1,2-Distearoyl-sn-glycero-3-phosphocholine; DSPE: 1,2-Distearoyl-sn-glycero-3-phosphoethanolamine; DSPE-PEG: 1,2-Distearoyl-sn-glycero-3-phosphoethanolamine-(polyethylene glycol)-2000; DSPE-PEG-SPDP: 1,2-Distearoyl-sn-glycero-3-phosphoethanolamine-[N-PDP(polyethylene glycol)-2000]; HSPC: Hydrogenated soy phosphocholine; PC: Phosphatidylcholine; pDNA: Plasmid DNA; PDP: 3-(2-Pyridyldithio)-Propionate; POPC: 1-Palmitoyl-2-oleoyl-sn-glycero-3-phosphocholine

1 Folate decorated nanocarrier for targeted delivery.

**Table 5:** Examples of recent studies on pH-sensitive liposomes

Stage of study	Cargo	*In-vivo* tumor model	Components	Targeting ligand	Reference
***In-vitro***	Docetaxel	_	PE, Chol, Oleic acid, Linoleic acid, CHEMS	_	(191)
***In-vitro***	DOX, Marker	_	SPC, Chol, DSPE-PEG, PEtOz-CHEMS	_	(192)
***In-vitro***	DOX, Marker	_	HSPC, DOPC, Chol, PEGm-PDPAn-PEGm	_	(193)
***In-vitro***	Paclitaxel	_	DOPE, DSPE-PEG, CHEMS	_	(194)
***In-vitro*** **/** ***In-vivo***	DOX, Marker	Colorectal cancer (HCT116 cells)	DPPC, mPEG-P(HPMA-g-His)-Col	_	(195)
***In-vitro*** **/** ***In-vivo***	Temsirolimus	Murine renal carcinoma (A498 cells)	SPC, Chol, a synthetic smart lipid (HHG2C18)	_	(196)
***In-vitro*** **/** ***In-vivo***	Paclitaxel	_	SPC, DSPE-PEG, CHEMS-PEG, CHEMS-Hz-PEG	_	(179)
***In-vitro*** **/** ***In-vivo***	miRNA, Marker	_	Chol, DMG-PEG, a pH-sensitive lipid	_	(197)
***In-vitro*** **/** ***In-vivo***	Ovalbumin, Marker	Mouse lymphoma (E.G7-OVA cells)	EPC, 3,5-Didodecyloxybenzamidine hydrochloride, MGlu-HPG	_	(174)
***In-vitro***	siRNA, Marker	_	DOPC, DODAP, N-dod-DOPE	Anti-CXCR4 antibodies	(182)
***In-vitro***	DOX, Marker	_	2IPC, DSPA, DSPE-PEG	Folate	(198)
***In-vitro*** **/** ***In-vivo***	Paclitaxel, Marker	Murine mammary carcinoma (4T1 cells)	SPC, Chol, DSPE-PEG, PEG5000-Hz-PE	R8 peptide	(178)
***In-vitro*** **/** ***In-vivo***	Paclitaxel, Marker	Murine melanoma tumor (B16F1 cells)	SPC, Chol, DSPE-PEG, DSPE-SS-PEG5000	TAT peptide	(199)
***In-vitro*** **/** ***In-vivo***	DOX	Breast adenocarcinoma (MDA-MB-231 cells)	DOPE, DSPE-PEG3400, CHEMS	Alendronate	(200)
***In-vitro*** **/** ***In-vivo***	Paclitaxel, Marker	Murine hepatocellular carcinoma (HepG2 cells)	SPC, Chol	CPP, Hyaluronic acid	(201)
***In-vitro*** **/** ***In-vivo***	DOX, Marker	Colon adenocarcinoma (HT29 cells)	SPC, Chol, DSPE-PEG	STP peptide	(202)
***In-vitro*** **/** ***In-vivo***	Paclitaxel, Marker	Murine melanoma (B16F10 cells)	SPC, Chol, DSPE-PEG	pH-responsive CPP and cRGD peptide	(203)
***In-vitro*** **/** ***In-vivo***	Paclitaxel, Marker	Colon adenocarcinoma (C26 cells)	SPC, Chol, DSPE-PEG, [D]-H6L9 peptide	cRGD peptide	(204)
***In-vitro/In-vivo***	DOX, Marker	Rat glioma (C6 cells)	Chol, DSPE-PEG, DOPE	pH-responsive CPP	(185)
***In-vitro/In-vivo***	DOX, Marker	Epidermoid carcinoma (KB cells)	PC, Chol, DSPE-PEG, DOTAP, Malachite green carbinol base	Folate	(205)
***In-vitro*** **/** ***In-vivo***	DOX	Breast carcinoma (MCF7 cells)	HSPC, DOPE, CHEMS, DSPE	Estrone	(189)

**Abbreviations:** CHEMS: Cholesteryl hemisuccinate; CHEMS-Hz-PEG: Cholesteryl hemisuccinate-Hidrazone activated-(polyethylene glycol)-2000 (An acid cleavable PEG-lipid derivative); CHEMS-PEG: Cholesteryl hemisuccinste-(polyethylene glycol)-2000; Chol: Cholesterol; CPP: Cell-penetrating peptide; cRGD peptide: Cyclic Arg-Gly-Asp peptide; CXCR4: C-X-C chemokine receptor type 4; [D]-H6L9 peptide: A pH-responsive anti-microbial peptide; DMG-PEG: 1,2-Dimyristoyl-sn-glycerol-methoxy(polyethylene glycol)-2000; DODAP: 1,2-Dioleoyl-3-dimethylammonium-propane; DOPC: 1,2-Dioleoyl-sn-glycero-3-phosphocholine; DOPE: 1,2-Dioleoylsn-glycero-3-phosphoethanolamine; DOTAP: 1,2-Dioleoyl-3-trimethylammoniumpropane; DOX: Doxorubicin; DPPC: 1,2-Dipalmitoyl-sn-glycero-3-phosphocholine; DSPE: 1,2-Distearoyl-sn-glycero-3-phosphoethanolamine; DSPE-PEG: 1,2-Distearoyl-sn-glycero-3-phosphoethanolamine-(polyethylene glycol)-2000; DSPE-SS-PEG: 1,2-Distearoyl-sn-glycero-3-phosphoethanolamine thiolytic cleavable-(polyethylene glycol)-2000; DSPA: 1,2-Distearoyl-sn-glycero-3-phosphate; EPC: Egg phosphatidylcholine; HHG2C18-L: (1,5-Dioctadecyl-l-glutamyl2-histidyl-hexahydrobenzoic acid-liposome) (A zwitterionic oligopeptide liposomes; HSPC: Hydrogenated soy phosphocholine; 2IPC: 1,2-diheneicosanoyl-sn-glycero-3-phosphocholine; MGlu-HPG: 3-Methylglutarylated hyperbranched poly(glycidol); miRNA: MicroRNA; mPEG-P(HPMA-g-His)-Chol: Methoxy-(polyethylene glycol)-b-poly(N-2-hydroxypropyl methacrylamide-co-histidine)-cholesterol; N-dod-PE: 1,2-Dioleoyl-sn-glycero-3-phosphoethanolamine-N-dodecanoyl; PEG5000-Hz-PE: (Polyethylene glycol)-5000-Hidrazone activated-Polyethylene; PC: Phosphatidylcholine; PE: Polyethylene; PEGm-PDPAn-PEGm: Di-block copolymer PEG8-PDPA15; PEtOz-CHEMS: Poly(2-ethyl-2-oxazoline)-cholesteryl hemisuccinate; R8 peptide: Octaarginine peptide; siRNA: Small (or short) interfering RNA; SPC: Soybean phosphatidylcholine; STP peptide: Dual-recognition (SKDEEWHKNNFPLSP) peptide; TAT peptide: (GRKKRRQRRRPQ) peptide.

**Table 6:** Examples of recent studies on enzyme-sensitive liposomes

**Stage of study**	**Cargo**	***In-vivo*** ** tumor model**	**Components**	**Type of enzyme**	**Targeting ligand**	**Reference**
*In-vitro*	Marker	_	DPPC, DMPC, DPPG, DMPG	Secretory phospholipase A2	_	(213)
*In-vitro*	_	_	DSPG, thio-ester pro anticancer ether lipid	Secretory phospholipase A2	_	(223)
*In-vitro*	DOX, Marker	_	DPPC, Poloxamer 188	Secretory phospholipase A2	_	(224)
*In-vitro/In-vivo*	Oxaliplatin	Breast cancer (MT3-cells)	POPC, POPG, Chol, DSPE-PEG	Secretory phospholipase A2	_	(212)
*In-vitro/In-vivo*	DOX, Marker	Prostate cancer (PC-3 cells)	DSPC, DSPG, Chol, DSPE, DSPE-PEG	Secretory phospholipase A2	_	(225)
*In-vitro*	Marker	_	POPC, Stearic acid conjugated collagen-mimetic peptides	MMP-9	_	(217)
*In-vitro*	Marker	_	DOPC, DSPC, POPC, MMP-9-sensitive lipopeptide	MMP-9	_	(216)
*In-vitro*	Marker		EPC, Chol, DSPE-PEG, DSPE-peptide-PEG3400	MMP-2	2C5 monoclonal antibody, TAT peptide	(215)
*In-vitro*	Marker	_	DOPE, DODAP, DOPE conjugated to elastase-sensitive peptide	Protease (Elastase)	_	(226)
*In-vitro*	pDNA	_	DOPE, DOTAP, PEG lipid with an enzymatically-cleavable linker	Cathepsin B	_	(221)
*In-vitro/In-vivo*	Paclitaxel conjugated to dendrimers by enzyme-sensitive linker, Marker	Breast cancer (MDA-MB-231 cells)	EPC, Chol, DSPE-PEG	Cathepsin B	Folate	(222)
*In-vitro/In-vivo*	siRNA, Marker	Prostate cancer (22Rv1 cells)	SPC, 3b[N-(N0 ,N0-dimethylaminoethane)-carbamoyl] chol, DSPE-PEG	PSA enzyme	Folate, CPPs	(219)

**Abbreviations: **Chol: Cholesterol; CPPs: Cell penetrating peptides; DMPC: 1,2-Dimyristoyl-sn-glycero-3-phosphocholine; DMPG: 1,2-Dimyristoyl-sn-glycero-3-phosphoglycerol; DODAP: 1,2-Dioleoyl-3-dimethylammonium-propane; DOPC: 1,2-Dioleoyl-sn-glycero-3-phosphocholine; DOPE: 1,2-Dioleoylsn-glycero-3-phosphoethanolamine; DOTAP: 1,2-Dioleoyl-3-trimethylammoniumpropane; DOX: Doxorubicin; DPPC: 1,2-Dipalmitoyl-sn-glycero-3-phosphocholine; DPPG: 1,2-Dipalmi-toyl-sn-glycero-3-phosphoglycerol; DSPC: 1,2-Distearoyl-sn-glycero-3-phosphocholine; DSPE: 1,2-Distearoyl-sn-glycero-3-phosphoethanolamine; DSPE-PEG: 1,2-Distearoyl-sn-glycero-3-phosphoethanolamine-(polyethylene glycol)-2000; DSPG: 1,2-Distearoyl-snglycero-3-phospho-(1'-rac-glycerol); EPC: Egg phosphatidylcholine; MMP-2: Matrix mettaloprotease-2; MMP-9: Matrix mettaloprotease-9; pDNA: Plasmid DNA; POPC: 1-Palmitoyl-2-oleoyl-sn-glycero-3-phosphocholine; POPG: 1-Palmitoyl-2-oleoyl-sn- glycero-3-phospho-(1’-rac-glycerol); PSA: Prostate-specific antigen; siRNA: Small (or short) interfering RNA; SPC: Soybean phosphatidylcholine; TAT peptide: (GRKKRRQRRRPQ) peptide

**Table 7:** Examples of recent studies on redox-sensitive liposomes

**Stage of study**	**Cargo**	***In-vivo*** ** tumor model**	**Components**	**Targeting ligand**	**Reference**
*In-vitro*	Marker	_	DOPE, Q-DOPE, DOPE-PEG	_	(240)
*In-vitro*	DOX, Marker	_	DSPC, DSPG, Ferrocene modified phospholipid prepared from DSPE and ferroceneacetic acid	_	(241)
*In-vitro*	Redox-responsive docetaxel prodrug, Marker	_	SPC, Chol, DSPE-PEG	_	(242)
*In-vitro*	Marker	_	POPC, POPE, Gallate derivative with three propargyl groups	_	(243)
*In-vitro*	DOX	_	Cerasome forming lipid with disulfide bond	_	(244)
*In-vitro*	pDNA, Marker	_	DOPE, Redox-sensitive gemini cationic Chol lipids	_	(245)
*In-vitro/In-vivo*	Paclitaxel, siRNA, Marker	Breast cancer (4T1 cells)	SPC, Chol, redox-sensitive cationic lipid	_	(234)
*In-vitro/In-vivo*	DOX	Murine osteosarcoma (MG63 cells)	SPC, Chol-SS-COOH, Chitooligosaccharides	_	(233)
*In-vitro/In-vivo*	Redox-responsive docetaxel prodrug	Lung carcinoma (A549 cells)	SPC, Chol, DSPE-PEG	_	(236)
*In-vitro*	DOX, Marker	_	EPC, DOPE, lipid like conjugate with disulfide bond and a biotin moiety	anti-HER2 antibody	(237)
*In-vitro*	Marker	_	DPPC, DOPE, CHEMS, DOPE-S-S-PEG	R8 peptide	(246)
*In-vitro/In-vivo*	DOX	Murine osteosarcoma (MG63 cells)	SPC, DOPE, DOTAP, Chol-SS-mPEG	Hyaluronic acid	(238)
*In-vitro/In-vivo*	Paclitaxel, Marker	Murine melanoma (B16F1)	SPC, Chol, DSPE-PEG, DSPE-SS- PEG5000	TAT peptide	(199)
*In-vitro/In-vivo*	Marker	Colon carcinoma (C26 cells)	EPC, Chol, Chol-S-S-PEG5000	R8 peptide	(239)
*In-vitro/In-vivo*	DOX, Verapamil, Marker	Breast cancer (MCF7 cells)	EPC, Chol, Chol-PEG2000, Chol-S-S-PEG5000	R8 peptide	(235)

**Abbreviations: **CHEMS: Cholesteryl hemisuccinate; Chol: Cholesterol; Chol-PEG: Cholesterol anchored modified-(polyethylene glycol)-2000; Chol-SS-COOH: Cholesterol anchored reduction-sensitive COOH; Chol-SS-PEG: Cholesterol anchored reduction-sensitive-(polyethylene glycol)-2000; DOPE: 1,2-Dioleoylsn-glycero-3-phosphoethanolamine; DOPE-PEG: 1,2-Dioleoylsn-glycero-3-phosphoethanolamine (polyethylene glycol)-2000; DOPE-SS-PEG: 1,2-Dioleoylsn-glycero-3-phosphoethanolamine anchored modified (polyethylene glycol)-2000; DOTAP: 1,2-Dioleoyl-3-trimethylammonium-propan; DOX: Doxorubicin; DPPC: 1,2-Dipalmitoyl-sn-glycero-3-phosphocholine; DSPC: 1,2-Distearoyl-sn-glycero-3-phosphocholine; DSPE: 1,2-Distearoyl-sn-glycero-3-phosphoethanolamine; DSPE-PEG: 1,2-Distearoyl-sn-glycero-3-phosphoethanolamine-(polyethylene glycol)-2000; DSPG: 1,2-Distearoyl-snglycero-3-phospho-(1'-rac-glycerol); EPC: Egg phosphatidylcholine; HER2: Human epidermal growth factor receptor 2; pDNA: Plasmid DNA; POPC: 1-Palmitoyl-2-oleoyl-sn-glycero-3-phosphocholine; POPE: Palmitoyl-oleoyl-phosphoethanolamine; Q-DOPE: Quinone-dioleoyl phosphatidylethanolamine; R8 peptide: Octaarginine peptide; siRNA: Small (or short) interfering RNA; SPC: Soybean phosphatidylcholine; TAT peptide: (GRKKRRQRRRPQ) peptide

To overcome limited uptake and specificity of stealth liposomes, some studies have been designed targeted pH-sensitive liposomes by conjugating various ligands such as antibodies (182, 183), peptides (178, 184 and 185), hyaluronic acid (186), transferrin (187), and folate (188) to the vesicle surfaces. Estrone decorated pH-sensitive liposomes were also designed for intracellular delivery of DOX to estrogen receptor on breast cancer cells (189). The estrogen receptor expression amplifies in breast carcinomas (190). Liposomes were prepared from DOPE, HSPC, CHEMS, and cholesterol. The targeted pH triggered formulation showed enhanced nuclear drug delivery, improved therapeutic efficacy, and reduced cardiotoxicity compared with non-triggered formulation and free drug (189).

*Enzyme-sensitive liposomes*

In some pathological conditions, such as cancer, inflammation, and infection, the concentrations of different extracellular and intracellular enzymes are elevated. Enzyme-responsive nanocarriers can be designed to undergo structural transformation and release the encapsulated cargoes by this biochemical abnormality (206, 207).

Enzyme-responsive liposomes have a number of advantages. The payload release is controlled by an enzyme at the target site without any external equipment for triggering. The amount of drug release in the targeted tissue is usually proportional to the concentration of the active enzyme and the severity of the pathological condition. Furthermore, some bioactive molecules are produced following enzyme digestion that may have synergistic therapeutic effects or facilitate the uptake of the drug. Secreted phospholipase A2, matrix metalloproteinases, urokinase plasminogen activator, elastase, and prostate-specific antigen are extracellular enzymes and cathepsin B is an intracellular enzyme used as triggers for drug release from liposomal carriers (206-208). Table 6 represents recent studies on enzyme-responsive liposomes. 

Secreted phospholipase A2 level increases in cancers (especially in the prostate, pancreatic, colon, and breast tumors), inflammatory diseases, cardiovascular diseases, and immune disorders (209). Therefore, phospholipase A2-responsive liposomes are attractive nanocarriers for the targeted release of anticancer agents at the tumor tissues.

Enzyme-mediated phospholipids hydrolysis disrupts the integrity of the lipid bilayer and triggers drug release. Involvement of phospholipase A2 receptor in the uptake of this responsive liposome has also been proposed. Another possible mechanism is cleavage of a lipophilic drug attached to the carrier by a phospholipase A2-sensitive bond (206, 210 and 211). A number of factors influence phospholipase A2 hydrolytic activity including the enzyme isoforms, lipid assembly, lipid physical properties, liposomal composition, and presence of lipopolymer. Incorporation of short acyl chain lipids, anionic polar head groups, and PEG grafted lipids have shown to increase the hydrolytic activity of phospholipase A2 (212, 213).

Matrix metalloproteinases (MMPs) are responsible for the proteolytic degradation of extracellular matrix. These enzymes (particularly, MMP-2 and MMP-9) are overexpressed in pancreatic, colorectal, breast, and lung tumors and play important roles in tumor growth, invasion, and metastasis (214). There are two main strategies to prepare MMP-responsive liposomes (206). First, MMP-sensitive peptides are synthetized and linked the shielding PEG groups to liposomal surface. At target site, the peptide is cleaved leading to the release of PEG and subsequently ligands promoting cellular uptake of nanocarriers are exposed to the target cancer cells. Torchilin and his coworkers designed a dual antinucleosome monoclonal antibody and TAT peptide targeted MMP-2-responsive multifunctional liposomal delivery system. Upon nanocarrier accumulation in tumors and specific targeting of cancer cells, in response to up-regulated extracellular MMP-2 in tumors, the hidden surface-attached TAT peptides exposed and enhanced cellular internalization of liposomes (Figure 8) (215). Another strategy is the incorporation of MMP-cleavable lipopeptides into the liposomal membrane. The lipopeptide cleavage in response to elevated enzyme concentration at the tumor tissues leads to liposomes destabilization and content release (216, 217).

The urokinase plasminogen activator and prostate-specific antigen are serine proteases. Urokinase plasminogen activator elevated levels have been reported in a variety of cancers including colon, bladder, breast, and ovarian tumors (218). Therefore, liposomes containing urokinase plasminogen activator cleavable peptides can release the encapsulated payloads at target sites. Prostate-specific antigen-activated nanoparticles developed from the enzyme-cleavable peptides can exhibit very selective antitumor activity against prostate cancer (219).

Cathepsin B is a lysosomal cysteine proteinase of the papain family enhanced extracellular matrix degradation and overexpressed in several malignancies, such as colon, prostate, brain, breast, and lung tumors (220). Cathepsin B has been reported to increase fusogenicity of liposomes at the target sites. Since cathepsin B is mainly found in lysosomes, dual pH and cathepsin B-responsive liposomes have been developed for targeted intracellular cargo delivery. In a recent article, PEG was attached to a lipid by an enzymatically-cleavable linker (glycine-phenylalanine-leucine-glycine). In the endosome, the detachment of PEG shell following the degradation of the peptide linker by cathepsin B caused vesicle destabilization, endosomal disruption, and triggering the controlled plasmid DNA release into cytoplasm (221). In another study, Satsangi *et al. *designed paclitaxel conjugated poly(amidoamine) dendrimers by cathepsin B-cleavable tetrapeptide and encapsulated this conjugate within folate receptor targeting liposomes (222).

*Redox-sensitive liposomes*

Redox-responsive delivery systems are one of the most efficient stimulus-responsive carriers for cancer drug and gene therapy. Glutathione (GSH) is a cysteine-containing tripeptide and the key intracellular reducing agent which plays an important role in cell growth and function as well as maintaining cellular redox homeostasis (227, 228). Much higher concentration of GSH (~100-1000 fold) in the intracellular compartments, especially in cytosol, mitochondria, and cell nucleus, compared to its levels in blood and extracellular matrix along with high redox potential difference existing between normal and tumor tissue provide a good rationale for redox-responsive nanocarriers as an intracellular drug delivery and tumor specific strategy. Typically, redox-sensitive nanostructures contain the cleavable/reversible disulfide bonds in their structures to render redox-responsive character (229-231). Redox-responsive liposomes have been often destabilized either by changes in hydrophilicity and/or charge of the amphiphile with reducing agents, or by cross-linker removal to cause lipid phase transitions (232). Table 7 summarizes recent studies on redox-responsive liposomes.

A reduction-sensitive fusogenic liposome was prepared by vesicle surface-coating with chotooligosaccharides, hydrolytic products of chitosan, via a disulfide linker (233). The hydrophilic backbone, low degree of polymerization, high water-solubility, cationic nature, cell adhesion properties, and wider biological activities such as anti-angiogenesis and radical scavenging efficacy make them as candidates for modification of tumor-targeted liposomes (233). Modified liposomes were stable under physiological conditions but destabilized in the presence of the cytosolic level of reducing agents most likely due to disulfide bond breakage. Chotooligosaccharide coated liposomes exhibited a prolonged half-life of DOX by 4-5.5 fold and strong inhibitory effect on tumor growth in osteosarcomas animal model compared to free drug (233). 

The survivin overexpression is an important factor involved in paclitaxel resistance of breast cancer cells. In a recent study, Chen *et al. *proposed redox-sensitive oligopeptide liposomes for co-delivery of paclitaxel and anti-survivin siRNA for the synergistic treatment and efficient anti-metastasis strategy against breast cancer (234). The nanosystem was composed of soybean phosphatidylcholine, cholesterol, and a redox-sensitive cationic oligopeptide lipid with a proton sponge effect. The vesicles disassembled in the presence of 10 mM GSH as confirmed by monitoring size, zeta potential, and morphology changes. The system offered several advantages including improved cellular uptake, reduced survivin expression, efficient endolysosomal escape, higher cell cytotoxicity, synergistic *in-vivo *inhibitory effect on tumor growth, and reduced pulmonary metastasis of breast cancer (234). Another co-delivery approach has been reported for combination of DOX and P-glycoprotein, verapamil, by a redox-responsive liposome to overcome multidrug resistance (235).

In contrast to studies utilized disulfide bonds in lipid components of vesicles, Ren *et al. *prepared a redox-responsive prodrug of docetaxel prodrug by conjugation of the drug molecule to vitamin E via a disulfide linker and incorporated it in liposomes (236).

A few studies have focused on designing targeted redox-responsive liposomal carriers by employing antibody (237), hyaluronic acid (238), and CPPs (199, 239). Cationic redox-sensitive liposomes were prepared with a novel detachable PEG conjugated with cholesterol through a disulfide linker and hyaluronic acid, a ligand for CD44, was non-covalently coated on the cationic vesicles. This nanosystem destabilized in reducing conditions and released higher cargo levels compared to redox insensitive liposomes. The proposed nanostructure was an excellent CD44-mediated intracellular delivery system for osteosarcoma treatment in animal models (238).

## Conclusion

Liposomes are a viable carrier to improve both the safety and efficacy of antineoplastic therapeutics which have already resulted in marketed anticancer products (*i.e.*, Doxil®, DaunoXome®, and Depocyt®) (247). To improve their efficacy and overcome the limitations of conventional liposomes, modified formulations have been investigated including stimuli-sensitive liposomes. This review evidences that numerous research efforts have been recently devoted to the optimization of liposomal carriers that allow delivering chemotherapeutics locally upon external as well as internal stimulation. Herein we have summarized the latest researches on stimuli-responsive liposomes. We also try to mention researches on combination of active targeting and active triggering for cancer therapy. Further attempts on industrialization are in great demand to bring these developments closer to oncology clinics.
